# Heterosexual anal intercourse and HIV infection risks in the context of alcohol serving venues, Cape Town, South Africa

**DOI:** 10.1186/1471-2458-11-807

**Published:** 2011-10-14

**Authors:** Seth C Kalichman, Steven D Pinkerton, Michael P Carey, Demetria Cain, Vuyelwa Mehlomakulu, Kate B Carey, Leickness C Simbayi, Kelvin Mwaba, Ofer Harel

**Affiliations:** 1 Department of Psychology, 406 Babbidge Road, University of Connecticut, Storrs, CT 06269, USA; 2 Center for AIDS Intervention Research, Medical College of Wisconsin, Milwaukee, Wisconsin, USA; 3 Center for Health and Behavior, Syracuse University, Syracuse, New York, USA; 4 Human Sciences Research Council, Cape Town, South Africa

## Abstract

**Background:**

The most efficient sexual behavior for HIV transmission is unprotected receptive anal intercourse. However, it is unclear what role heterosexual unprotected anal sex is playing in the world's worst HIV epidemics of southern Africa. The objective is to examine the prevalence of heterosexual unprotected anal intercourse among men and women who drink at informal alcohol serving establishments (shebeens) in South Africa.

**Methods:**

Cross-sectional surveys were collected from a convenience sample of 5037 patrons of 10 shebeens in a peri-urban township of Cape Town, South Africa. Analyses concentrated on establishing the rates of unprotected anal intercourse practiced by men and women as well as the factors associated with practicing anal intercourse.

**Results:**

We found that 15% of men and 11% of women reported anal intercourse in the previous month, with 8% of men and 7% of women practicing any unprotected anal intercourse. Multiple logistic regression showed that younger age, having primary and casual sex partners, and meeting sex partners at shebeens were independently associated with engaging in anal intercourse. Mathematical modeling showed that individual risks are significantly impacted by anal intercourse but probably not to the degree needed to drive a generalized HIV epidemic.

**Conclusions:**

Anal intercourse likely plays a significant role in HIV infections among a small minority of South Africans who patronize alcohol serving establishments. Heterosexual anal intercourse, the most risky sexual behavior for HIV transmission, should not be ignored in HIV prevention for South African heterosexuals. However, this relatively infrequent behavior should not become the focus of prevention efforts.

## Background

The prevalence of anal intercourse among heterosexual adults has not been well-studied in many populations. This knowledge gap is important because, relative to vaginal intercourse, anal intercourse may be as much as 20 times more efficient for transmitting HIV. Given the transmission efficiency of anal intercourse, even a low prevalence of anal sex among heterosexuals could account for a large number of HIV infections [1].

Estimates from the U.S. and U.K. suggest an average heterosexual anal intercourse prevalence of 11% (range = 2% to 39%) [1,2]. The highest rates have been found among female sex workers and patients at sexually transmitted infections (STI) clinics [3]. As many as 20% of STI clinic patients in South Africa report engaging in unprotected anal intercourse with their most recent partner [4]. It is also noteworthy that, among heterosexuals, anal intercourse often co-occurs with other risky sexual practices (e.g., multiple and concurrent sex partners, selling/trading sex, using alcohol and other drugs), increasing risk further [3,5].

The role of anal intercourse in southern Africa - where the greatest concentration of heterosexually-transmitted HIV cases are found - remains under-investigated. Studies of specific population sub-groups are most common. For example, research has shown that 43% of female sex workers [6] and 20% of truck drivers [7] report lifetime anal intercourse. In addition, 14% of men and 10% women recruited from South African STI clinics and impoverished communities reported engaging in anal intercourse in the past three months [8]. Among individuals reporting anal intercourse, 28% engaged in this behavior more often than vaginal intercourse. As occurs elsewhere, anal intercourse in South Africa tends to occur in the context of other high-risk factors, including substance use. However, focusing on sex workers and persons who have contracted an STI may overestimate the prevalence of anal intercourse.

Prevalence estimates of anal intercourse can help interventionists effectively tailor prevention messages. HIV prevention programmers must decide the degree to which messaging should emphasize anal intercourse relative to other risk behaviors; the former may be less prevalent but more efficient in transmitting HIV. Ignoring anal intercourse in prevention messages may suggest it is unimportant. On the other hand, overemphasizing a low-base rate behavior in prevention messages may inadvertently reinforce the idea that vaginal intercourse is low risk for HIV transmission. Determining the appropriate balance of attention to relative risks is best guided by behavioral surveillance research of targeted risk practices.

An important group for targeted HIV prevention in South Africa is persons who patronize informal alcohol serving establishments (shebeens). Shebeens are common throughout southern Africa and, importantly, are a meeting place for sex partners [9-11]. Among men and women recruited from informal local shebeens, more than one in four report having met sex partners at a shebeen [12]. Individuals who meet sex partners in shebeens have greater numbers of recent sex partners and higher rates of unprotected intercourse compared to persons who did not meet partners in shebeens [13]. The high frequency and heavy drinking in HIV prevalent places where sex partners are met creates significant risks for the spread of HIV. Thus, informal drinking venues and their surrounding communities are a high-priority for HIV prevention interventions in South Africa [9,14-16].

Given that shebeens provide an environment where sexual partnerships are formed among patrons who are likely to be infected with HIV, [17,18] this study focused on the prevalence of anal intercourse in this context. We hypothesized that anal intercourse would be observed at relatively low frequencies among a minority of persons, and that individuals who engaged in anal intercourse would report other high risk practices; a pattern of multiple risk behaviors would suggest that such persons may be at highest risk for HIV infection. We also used mathematical modeling techniques to estimate the increased risk for HIV acquisition conferred by unprotected anal intercourse to women who drink at shebeens.

## Methods

### Participants

Participants were 5037 residents (3444 men, 1593 women) in ten sections of a primarily Xhosa-speaking township outside Cape Town, South Africa. All participants were age 18 or older (median = 30 years old). Nearly all (98%) participants identified as Black African, 53% were unemployed, and 50% had not matriculated school.

### Research setting and procedures

The study occurred in a township located 20 km outside of Cape Town's central business district. Residents are primarily of Xhosa heritage. Neighborhoods were defined as an area ½ km wide that contained at least one shebeen. Using methods described by Weir et al. [11,19], we conducted rapid community assessments to identify 10 shebeens separated by ≥ 1 km. All shebeens were visited and we interviewed owners, managers, and patrons to confirm that the shebeens served ≥ 75 patrons per week. Surveys were conducted by 8 indigenous field workers who spoke both Xhosa and English. The field workers approached persons on the street and individuals socializing and drinking in the shebeens, and asked them if they would complete an anonymous, self-administered survey. Individuals who agreed to participate in the study (95%) were given a survey that most completed within 20 minutes. Participants were compensated for their time with a non-monetary item (keychain or shopping bag). Surveys were collected inside (48% of men and 37% of women) and outside (52% of men and 63% of women) of the shebeens. All procedures were approved by the Institutional Review Boards of the University of Connecticut, Syracuse University, and the Human Sciences Research Council or South Africa.

### Measures

Participants were asked to report demographic characteristics, shebeen attendance, HIV risk history and sexual behaviors including drinking alcohol in conjunction with sex.

#### Demographic characteristics

Participants reported their age, race, cultural heritage, education, marital and employment status. Participants also indicated whether they had been tested for HIV, their most recent test result, and STI treatment history.

#### Shebeen attendance

Participants were given a list of all the shebeens in their township section (as well as a response choice for "any other shebeen") and asked how many times they went to these shebeens in the past month; response options were (a) never, (b) 1 to 4 times, (c) 5 to 10 times, (d) 11 to 20 times or (e) 21 or more times.

#### Sexual risk behaviors

Participants reported the number of male and female sex partners they had in the past month and their number of specific sex acts (vaginal and anal intercourse with and without condoms). We calculated the percent of intercourse occasions protected by condoms for vaginal and anal intercourse separately. We also asked how many times in the past month participants drank alcohol before sex and how many times they had a partner who drank alcohol before sex. We selected a one-month timeframe with open response formats to improve recall accuracy and provide unanchored responses [20].

### Data analyses

Seventy-two men reported sex with male partners in the previous month. Of these men, 41 (56%) reported only male partners and 31 (44%) reported male and female partners; 46 (65%) of these men reported unprotected anal intercourse. Consistent with our primary research question, and to avoid confounding homosexual with heterosexual anal intercourse, we removed these 72 men from further analyses.

We performed descriptive analyses to examine the demographic characteristics, alcohol use, and sexual practices of men and women who engaged in anal intercourse versus those who did not. Preliminary comparisons of persons surveyed inside and outside of shebeens indicated few differences. Data collected inside and outside shebeens were therefore collapsed. We defined risk as engaging in any anal intercourse (because of the tendency toward inconsistent condom use and the potential for condom failures). We first compared men and women on the frequency of engaging in sexual risk behaviors using logistic regressions. We also performed logistic regression analyses to test the hypothesis that multiple independent behaviors would cluster to predict anal intercourse. The model simultaneously tested non-overlapping but significant predictors of anal intercourse from the previous analyses. Results from the logistic regressions are reported as odds ratios (OR) with 95% confidence intervals (CI). All analyses defined statistical significance as *p *< .05.

We also performed a modeling exercise to estimate the increased risk for HIV acquisition conferred by unprotected anal intercourse to women in our sample. We focused on women because receptive anal intercourse is the highest risk behavior for HIV transmission and because differences in the per-act transmission probabilities for insertive anal intercourse and insertive vaginal intercourse are believed to be minimal.

In these analyses a Bernoullian model of HIV transmission [21] was used to estimate female study participants' risk of HIV acquisition:

$$\text{risk }=\;1\;-\left[\left(1\;-\pi \right)\;+\pi {\left(1\;-\alpha_{V}\right)}^{n1}{\left(1\;-\left(1\;-\varepsilon \right)\alpha_{V}\right)}^{n2}{\left(1\;-\alpha_{A}\right)}^{n3}{\left(1\;-\left(1\;-\varepsilon \right)\alpha_{A}\right)}^{n4}\right]m,$$

where *m *denotes the number of sex partners and *n*^1^, *n*^2^, *n*^3^, and *n*^4^, denote the average number of acts, per partner, of unprotected vaginal intercourse, condom-protected vaginal intercourse, unprotected anal intercourse, and protected anal intercourse, respectively. The following parameter values were used in the base-case analysis: per-act transmission probability for receptive vaginal intercourse, α_V _= 0.003 [varied from 0.0014 to 0.0063 [3,22,23]. in the sensitivity analyses]; prevalence of HIV among sex partners, π = 10% (5% to 20%); and condom effectiveness, ε = 90% (0 to 100%). The per-act transmission probability for receptive anal intercourse was assumed to be 5.7 (2.1 to 14.1) times larger than the transmission probability for receptive vaginal intercourse. Therefore, the per-act transmission probability for receptive anal intercourse ranged from α_A _= 0.003 to 0.089, with a base-case value of 0.017, consistent with a recent meta-analysis [22].

Data from the study were used to estimate the average risk of HIV acquisition for study participants who engaged in anal intercourse (risk_A_) and the average risk of HIV acquisition for participants who did not engage in this activity (risk_B_). A weighted average was then calculated by multiplying these risk estimates by the proportion of study participants who fell into each of the corresponding categories (p_A _and p_B_): average risk_1 _= p_A_*risk_A _+ p_B_*risk_B_. An additional "hypothetical" risk estimate (risk_C_) was calculated by replacing all acts of receptive anal intercourse with vaginal intercourse. A second weighted average was then calculated using the hypothetical estimate: average risk_2 _= p_A_*risk_C _+ p_B_*risk_B_. The ratio of the weighted averages, *r *= average risk_1_/average risk_2 _is an indicator of the amount by which anal intercourse increased participants' risk of HIV acquisition. This ratio also can be translated into an estimate of the risk reduction (1 - 1/*r*) that would be achieved if all acts of anal intercourse were replaced by vaginal intercourse acts.

## Results

Among the 4,965 study participants, results showed that 88% of men and 84% of women reported at least one sex partner in the previous month, with 38% of men and 21% of women reporting two or more sex partners (see Table 1). Anal intercourse (previous month) was reported by 15% of men and 11% of women, with 8% of men and 7% of women reporting anal intercourse without condoms. Comparisons of men and women on other sexual behaviors (previous month) showed that men had more sex partners and engaged in more acts of vaginal and anal intercourse. Men and women differed in their condom use during vaginal and anal intercourse, with men reporting more condom use.

**Table 1:** Sexual practices in the previous month among men and women living in a Cape Town township

	Men			Women				
	(*n *= 3372)			(*n *= 1593)				
Behavior	Median	Mean	SD	Median	Mean	SD	OR	95% CI
Sex partners	1	1.9	4.5	1	1.3	1.9	1.13*	1.09-1.18
Unprotected vaginal intercourse	3	6.5	10.7	2	5.9	8.3	1.07	1.00-1.01
Vaginal intercourse with condoms	3	6.4	15.6	2	5.4	9.3	1.01**	1.00-1.02
Total vaginal intercourse	10	12.8	23.1	8.5	11.3	13.6	1.01**	1.00-1.01
Percent condom use								
during vaginal intercourse	50	49.6	41.0	50	49.2	41.4	1.02	0.87-1.20
Unprotected anal intercourse	0	0.5	3.3	0	0.4	3.0	1.01	0.99-1.03
Anal intercourse with condoms	0	0.8	3.5	0	0.4	3.2	1.06**	1.03-1.09
Total anal intercourse	0	1.3	5.9	0	0.8	5.7	1.03**	1.01-1.04
Percent condom use								
during anal intercourse	71	61.3	41.3	44	48.9	44.2	2.20**	1.47-3.28
		
	No.		%	No.		%		
		
Number of sex partners								
0	385		12	251		16		
1	1648		49	1001		63		
2	700		21	179		11		
3+	610		17	155		10	1.37**	1.29-1.46
Any unprotected vaginal intercourse	2023		61	901		57	1.14**	1.04-1.32
Any unprotected anal intercourse	278		8	118		7	1.13	0.90-1.42
Any anal intercourse	510		15	182		11	1.38**	1.15-1.65

All sexual behaviors measured for the past month; No. = number; M = mean; SD = standard deviation; OR = odds ratio; CI = confidence interval ** p < .01, * p < .05.

### Factors associated with engaging in anal intercourse

Table 2 shows the characteristics of participants who did and did not engage in anal intercourse, separately for men and women. For men, engaging in anal intercourse was associated with being unmarried and having primary, casual, and multiple recent sexual partners. Men who engaged in anal intercourse were also more likely to have met sex partners in shebeens in the past month. In addition, practicing anal intercourse was related to drinking before sex and having a partner who drank before sex. Men who engaged in anal intercourse were also more likely to have been diagnosed with an STI. For men, there were no associations between anal intercourse and HIV testing, or HIV status.

**Table 2:** Characteristics of men and women who did not and who did engage in anal intercourse in the previous month

	Men						Women					
										
	Did not engage	Did engage in					Did not engage	Did engage in				
	in anal sex (*n *= 2862)	anal sex (*n *= 510)					in anal sex (*n *= 1411)	anal sex (*n *= 182)				
Characteristic	**No**.	%	**No**.	%	OR	95%CI	**No**.	%	**No**.	%	OR	95%CI
Married	687	24	79	16	1.7**	1.20-2.10	298	21	38	21	1.01	0.69-1.48
Primary partnered	2113	84	435	89	1.65**	1.21-2.24	100	80	153	88	1.86**	1.14-3.06
Casual partners	1057	37	324	64	2.95**	2.43-3.60	248	18	67	36	2.71**	1.92-3.78
3+ sex partners	470	16	140	28	1.91**	1.53-2.38	129	10	26	14	1.64*	1.04-2.59
Met partners at												
shebeens in past month	498	18	164	32	2.24**	1.82-2.77	128	9	47	26	3.54**	2.42-5.17
STI	1053	37	285	56	2.16**	1.78-2.61	457	33	104	57	2.76**	2.02-3.78
HIV tested	1846	65	342	67	1.11	0.91-1.36	1052	75	143	79	1.30	0.89-1.90
HIV positive	138	7	26	7	1.15	0.96-1.37	135	12	13	8	1.30	1.00-1.71

No. = number; OR = odds ratio; CI = confidence interval.

Women showed a similar pattern of results. Women who engaged in anal intercourse were more likely to have primary, casual, and multiple recent partners. In addition, women who engaged in anal intercourse were more likely to have met sex partners in shebeens and to have had an STI.

Analyses showed that men who engaged in anal intercourse had more sex partners and engaged in more vaginal intercourse with and without condoms (Table 3). For women, engaging in anal intercourse was also associated with vaginal intercourse (condom protected and total). Among participants who engaged in anal intercourse in the past month, men had higher rates of anal intercourse with condoms than women. For both men and women engaging in anal intercourse was associated with alcohol use before sex as well as their partner's use of alcohol before sex.

**Table 3:** Sexual behaviors among men and women who did not and who did engage in anal intercourse in the previous month

	Men						Women					
										
	Did not engage in anal sex (*n *= 2862)	Did engage in anal sex (*n *= 510)					Did not engage in anal sex (*n *= 1411)	Did engage in anal sex (*n *= 182)				
Behavior	M	SD	M	SD	OR	95%CI	M	SD	M	SD	OR	95%CI
Number of partners	1.8	4.6	2.7	3.7	1.04**	1.01-1.08	1.3	1.9	1.6	1.3	1.05	0.98-1.12
Unprotected vaginal intercourse	6.1	8.2	8.3	19.3	1.02**	1.00-1.03	5.6	7.8	8.6	10.9	1.03	1.01-1.05
Vaginal intercourse with condoms	5.7	8.0	10.1	35.1	1.03**	1.02-1.04	5.1	7.9	7.3	16.4	1.01**	1.00-1.03
Total vaginal intercourse	11.6	11.4	18.3	52.2	1.03**	1.02-1.04	10.7	11.6	15.9	23.6	1.02**	1.01-1.03
Percent condom use during												
vaginal intercourse	48.3	41.4	55.7	38.4	1.55**	1.22-1.96	46.2	39.0	49.7	41.7	0.87	0.55-1.20
Unprotected anal intercourse^a^	3.5	7.9					3.7	8.4			0.99	0.97-1.01
Anal intercourse with condoms^a^	5.2	7.4					3.5	8.9			1.04**	1.01-1.08
Total anal intercourse^a^	8.7	12.9					7.2	15.7			1.01**	0.99-1.03
Percent condom use during												
anal intercourse^a^	61.3	41.3					46.9	44.2			2.20**	1.47-3.28
Alcohol use before sex	4.1	8.3	5.9	7.1	1.02**	1.01-1.03	2.3	5.6	10.0	89.0	1.02*	1.00-1.04
Partner drank before sex	1.4	4.9	2.6	4.7	1.04**	1.02-1.06	3.0	5.9	5.1	11.6	1.03**	1.01-1.05

AI = anal intercourse; ^a ^comparison of men and women who did engage in anal intercourse; ** p < .01, * p < .05.

### Multivariate analysis

To test independent predictors of anal intercourse, we conducted a simultaneous logistic regression with anal intercourse in the past month entered as the dependent variable and non-redundant risk behaviors entered as predictors. Results of the multiple logistic regression showed that younger age, having primary and casual sex partners, and meeting partners in shebeens were associated with anal intercourse (Table 4).

**Table 4:** Multivariable model predicting engaging in anal intercourse in the past month

Characteristic	OR	95% CI
Gender	1.15	0.93-1.41
Age	0.97**	0.96-0.98
Primary partners	1.56**	1.19-2.05
Casual partners	2.33**	1.92-2.83
Multiple partners	1.01	0.81-1.28
Met partners at		
shebeens in past month	1.81**	1.47-2.22
Alcohol use before sex	1.00	0.99-1.01
Partner drank before sex	1.01	0.99-1.03

OR = odds ratio; CI = confidence interval. ** p < .01

### Modeling HIV acquisition

The results of the mathematical modeling indicated that--across the values of the per-act transmission probabilities considered in the main analyses--women who engaged in receptive anal intercourse faced a 2.4 to 8.9 times greater risk of acquiring HIV than did sexually-active women who did not engage in this practice (base-case value = 4.4). However, much of the increase in risk was due to the greater numbers of unprotected intercourse acts reported by women who engaged in anal intercourse. Even after replacing all acts of anal intercourse by vaginal intercourse, these women would still face a 1.8 times greater risk of acquiring HIV.

Taking into account the fact that only a relatively small proportion of the women in the study reported anal intercourse, replacing all acts of anal intercourse with vaginal intercourse would reduce the mean sample-wide risk of HIV acquisition by approximately 24% (7% to 46%). (The estimates obtained in the sensitivity analyses, in which the values of the HIV prevalence and condom effectiveness parameters were varied independently and jointly, all fell within the approximate range observed in the main analyses).

## Discussion

In this study of shebeen patrons, 15% of men and 11% of women reported engaging in any anal intercourse in the previous month; 8% of men and 7% of women reported not using condoms during anal intercourse in the past 30 days. Anal intercourse was far less frequent than vaginal intercourse and condom use was more common during anal sex than vaginal sex. These findings confirm our main study hypothesis by demonstrating a small number of people engage in infrequent anal intercourse.

This is the first study to examine the relative rates of anal and vaginal intercourse among men and women who drink in informal alcohol serving venues in South Africa. Previous studies in South Africa report 43% of female sex workers [24] and 5% of adolescents [25] engaged in anal intercourse in their lifetime. STI clinic patients demonstrate similar frequencies of anal intercourse (unprotected and total) as those identified in this sample of shebeen patrons [8]. Thus, we found that anal intercourse likely contributes to the overall risks for HIV infection for a small number of people in high-risk drinking environments.

We also confirmed our second hypothesis, showing that anal intercourse occurs in the context of other sexual risk behaviors that create a pattern of high risk for HIV infection. People who practiced anal intercourse also reported more sex partners; they were also more likely to have had primary and casual sex partners. Engaging in anal intercourse was also associated with a history of STI and consuming alcohol in sexual contexts as well as one's partner drinking in sexual contexts. Overall, these findings suggest that targeting a broad array of risk factors for HIV transmission, such as having recent multiple sex partners, will likely also capture individuals who engage in anal intercourse. Given the stigma associated with anal intercourse [26,27], it may prove beneficial to reach the relatively small number of persons who practice this behavior by casting a broader net to reach people who engage in a cluster of risk practices.

The results of the modeling indicated that engaging in receptive anal intercourse increased female participants' average risk of acquiring HIV by a factor of 2.4 (range = 1.3. to 4.9). Replacing all acts of anal intercourse with vaginal intercourse acts would reduce the mean sample-wide risk of HIV acquisition by approximately 24% (range = 2% to 46%). Thus, the small proportion of women who engaged in anal intercourse disproportionately affected the mean risk of the sample as a whole. Therefore, reductions in unprotected anal intercourse could have a significant impact on individuals who practice this behavior and a small impact on HIV epidemics.

The current findings should be interpreted in light of the study limitations. We relied on self-reports of sexual behavior and substance use, behaviors that are private and socially stigmatized. Thus, for example, it is possible that some men did not report same-sex relationships and were included in the sample. It is also possible that participants confused the meaning of vaginal and anal sex, mistakenly reporting (or not reporting) the occurrence of anal sex. It is also possible that some participants mistakenly reported rear-entry vaginal intercourse as anal intercourse and vice versa. We also relied on a recall period of one month which can result in missed events falling outside this time frame, underestimating frequencies of behavior. The current study did not assess motivations for practicing anal intercourse, such as to maintain virginity or to avoid pregnancy. Our samples were also drawn by convenience and cannot be considered representative of Cape Town shebeens. Finally, our study requires replication and confirmation before drawing conclusions. Despite these limitations, we believe that our findings have important implications for HIV prevention in South Africa.

Unprotected anal intercourse substantially increases the risks for HIV transmission and may account for a portion of HIV infections in generalized epidemics. Consistent with other modeling studies [22], female participants who engaged in unprotected anal intercourse demonstrated a substantial increase in HIV acquisition risk relative to women who only practiced vaginal intercourse. Ignoring anal intercourse in HIV prevention interventions will miss important opportunities to prevent infections. At the same time, we caution against over-emphasizing the role of anal intercourse in generalized epidemics. Individuals who are at risk for HIV infection often gravitate toward underestimating their personal risk [28]. Misinterpreting these results (i.e., concluding that only heterosexuals who practice anal sex are at risk for HIV) could lead to false impressions of safety, with grave unintended consequences.

## Conclusions

Anal intercourse carries the highest risk for sexual transmission of HIV Infection. Unprotected anal intercourse is practiced at low-frequencies (mean 3.6 acts in the previous month) by 8% of men and 7% of women who drink at informal alcohol serving venues in Cape Town South Africa. Therefore a relatively small number of persons in South Africa's generalized AIDS epidemic practice this high-risk behavior. Targets for risk reduction interventions that aim to reduce unprotected anal sex include individuals who are younger, have primary and casual sex partners, and meet sex partners in drinking places engage in more anal intercourse. Anal intercourse may therefore play a significant role in HIV infections among a small minority of South Africans who patronize alcohol serving establishments.

## Key Points

•Among the men and women sampled from Cape Town drinking establishments, 8% engaged in unprotected anal intercourse, and they practiced this behavior at low-frequencies (mean 3.6 acts in the previous month).

•Individuals who are younger, have primary and casual sex partners, and meet sex partners in drinking places engage in more anal intercourse.

•Modeling shows that individual risks are significantly impacted by anal intercourse but not to a degree that would probably drive a generalized HIV epidemic.

•Anal intercourse may play a significant role in HIV infections among a small minority of South Africans who patronize alcohol serving establishments.

## Competing interests

The authors declare that they have no competing interests.

## Authors' contributions

SCK, conceptualized and directed the study, contributed to the data analysis, and was the lead writer. SDP, performed the mathematical modeling and contributed to the writing of the paper. MPC, contributed to the study conceptualization and writing the paper. DC, served as Co-Project Manager, oversaw scientific execution of study, contributed to writing the paper. VM, served as Co-Project Manager, oversaw scientific execution of study. KBC, contributed to the study conceptualization and writing the paper. LCS, contributed to the study conceptualization. KM, contributed to the study conceptualization. OH, contributed to the study conceptualization and data analyses. All authors read and approved the final version.

## Pre-publication history

The pre-publication history for this paper can be accessed here:

http://www.biomedcentral.com/1471-2458/11/807/prepub
